# *Aedes* (*Stegomyia*) *albopictus* (Skuse): A Potential Vector of Zika Virus in Singapore

**DOI:** 10.1371/journal.pntd.0002348

**Published:** 2013-08-01

**Authors:** Pei-Sze Jeslyn Wong, Mei-zhi Irene Li, Chee-Seng Chong, Lee-Ching Ng, Cheong-Huat Tan

**Affiliations:** 1 Environmental Health Institute, National Environment Agency, Singapore, Singapore; 2 School of Biological Sciences, Nanyang Technological University, Singapore, Singapore; United States Army Medical Research Institute of Infectious Diseases, United States of America

## Abstract

**Background:**

Zika virus (ZIKV) is a little known arbovirus until it caused a major outbreak in the Pacific Island of Yap in 2007. Although the virus has a wide geographic distribution, most of the known vectors are sylvatic *Aedes* mosquitoes from Africa where the virus was first isolated. Presently, *Ae. aegypti* is the only known vector to transmit the virus outside the African continent, though *Ae. albopictus* has long been a suspected vector. Currently, *Ae. albopictus* has been shown capable of transmitting more than 20 arboviruses and its notoriety as an important vector came to light during the recent chikungunya pandemic. The vulnerability of Singapore to emerging infectious arboviruses has stimulated our interest to determine the competence of local *Ae. albopictus* to transmit ZIKV.

**Methodology/Principal Findings:**

To determine the competence of *Ae. albopictus* to ZIKV, we orally infected local mosquito strains to a Ugandan strain virus. Fully engorged mosquitoes were maintained in an environmental chamber set at 29°C and 80–85%RH. Twelve mosquitoes were then sampled daily from day one to seven and on day 10 and 14 post infection (pi). Zika virus titre in the midgut and salivary glands of each mosquito were determined using tissue culture infectious dose_50_ assay, while transmissibility of the virus was determined by detecting viral antigen in the mosquito saliva by qRT-PCR. High dissemination and transmission rate of ZIKV were observed. By day 7-pi, all mosquitoes have disseminated infection and 73% of these mosquitoes have ZIKV in their saliva. By day 10-pi, all mosquitoes were potentially infectious.

**Conclusions/Significance:**

The study highlighted the potential of *Ae. albopictus* to transmit ZIKV and the possibility that the virus could be established locally. Nonetheless, the threat of ZIKV can be mitigated by existing dengue and chikungunya control program being implemented in Singapore.

## Author Summary

The prominence of *Ae. albopictus* as a major vector came to light during the global pandemic of chikungunya. The continual global expansion of *Ae. albopictus* coupled with data from competence studies that show its ability to transmit more than 20 arboviruses has fuelled growing concerns that this mosquito may alter the epidemiology of arbovirus worldwide and increase the risks of humans to mosquito-borne diseases. This has stimulated our interest to determine the extent of pathogens *Ae. albopictus* can transmit, especially arboviruses with high epidemic potential. The current study showed that local *Ae. albopictus* are susceptible to ZIKV. By day 7 pi. all mosquitoes that were orally infected were found to have disseminated infections and 73% have ZIKV in their saliva. This study highlighted the potential of *Ae. albopictus* to transmit ZIKV. The local transmission of ZIKV in Singapore is plausible due to the presence of susceptible vectors, an immunologically naive and vulnerable human population, and the travel hub status of Singapore. Nonetheless, the threat of ZIKV can be mitigated by existing dengue and chikungunya control program being implemented in Singapore.

## Introduction

The Asian tiger mosquito, *Aedes (Stegomyia) albopictus* (Skuse), is considered a vector or potential vector of several pathogens of human and veterinary importance. Viral isolation and vector competence studies have shown that this mosquito is an efficient vector of more than 20 arboviruses [1]. Due to its biological and ecological plasticity, this notoriously invasive species has a wide geographic distribution. At present, aside from its tropical Asian home, *Ae. albopictus* can be found in temperate Asian countries, in tropical and temperate Americas, Europe, Middle East, the Pacific islands, Australia and Africa [2], [3], [4], [5]. The global spread of *Ae. albopictus* is mainly caused by human activities, such as increase in intercontinental trade, especially in the last three decades [1].

In places where *Ae. aegypti* and *Ae. albopictus* co-exist, *Ae. albopictus* was considered second only to *Ae. aegypti* in terms of importance as vector of dengue and chikungunya [6], [7]. However, its notoriety as an important vector came to light during the recent unprecedented global outbreak of chikungunya. According to Tsetsarkin et al. [8], a mutation in the envelope gene of chikungunya virus (CHIKV) enhances the virus infectivity and transmissibility in *Ae. albopictus*. The continual global expansion of *Ae. albopictus* is a growing concern as this mosquito may alter the transmission dynamics of arboviral diseases and increase the risks of humans to mosquito-borne viral infections [3], [9]. This has stimulated increased interest to determine the extent of pathogens this mosquito can transmit.

Zika virus (ZIKV), a little known arbovirus, has gained prominence when it caused a large scale epidemic in the Pacific Island in 2007 [10], [11]. It is a member of the genus *Flavivirus* of the Family Flaviviridae [12]. The virus is a positive single stranded RNA virus with a 10,794 nucleotide genome that is closely related to the Spondweni virus [13], [14], [15]. It was first isolated from a febrile sentinel monkey in Uganda in 1947 [15], but human ZIKV infection was first reported in 1964 [16]. The virus causes dengue-like syndromes such as rash, fever, arthralgia, headache and peri-orbital pain [11], [16].

To date, only *Aedes* mosquitoes have been known to transmit ZIKV. In Africa, the virus was isolated from both sylvatic and peri-domestic mosquitoes: *Ae. africanus*, *Ae. apicocoargenteus*, *Ae. luteocephalus*, *Ae. furcifer*, *Ae. vitattus* and *Ae. aegypti* [17], [18], [19], [20], [21], [22]. In Asia, ZIKV was only isolated from a pool of *Ae. aegypti* caught from shop houses in the State of Pahang in Peninsular Malaysia [22]. During the ZIKV outbreak in Yap Island in 2007, no virus was isolated from any of the mosquitoes caught. However, based on epidemiological evidences, *Ae. hensilii* was suspected to be the vector responsible for the outbreak [11]. In 2010, a case of ZIKV involving a three year old child was reported in Kampong Speu Province in Cambodia, however, the vector responsible was not known [23].

Identification of vectors and potential vectors of ZIKV or any other mosquito-borne diseases in a given geographical area has important implication when it comes to disease outbreak control. It is imperative that vectors are identified, so that a holistic and sound vector control program can be formulated. To date, *Ae. aegypti* is the only vector of ZIKV identified in Southeast Asia [22] and data on mosquito-ZIKV interactions have also been confined to this mosquito [24], [25]. Our recent study has also showed that local *Ae. aegypti* strains are highly susceptible to ZIKV and viral dissemination rates reflect that observed for a local, highly epidemic DENV-2 strain [26].

In the light of continuous global niche expansion of *Ae. albopictus*, coupled with its catholic feeding behaviour, ecological adaptability and propensity to support a wide range of arboviruses, it is important to determine its competence to transmit pathogens with high epidemic potential such as ZIKV. The current study describes ZIKV infection in our local *Ae. albopictus*.

## Materials and Methods

### Mosquitoes

*Aedes albopictus*, used for the experimental infection, was derived from eggs collected during weekly ovitrap surveillance study as previously described [26]. Mosquitoes were colonized under standard insectary conditions as described by Li and colleagues [26]. In order to obtain enough number of mosquitoes for the study, F_3_ generations were used.

### Virus

Ugandan MR766 ZIKV strain obtained from the American Type Culture Collection (Manassas, VA, USA) was used to infect the mosquitoes. The stock virus used in the current study was passaged thrice in Vero cell line prior to the infectious feed [26].

### Oral infection of mosquitoes

Five to seven-day old female mosquitoes (*n* = 120) were transferred to 0.5 L containers and starved for 24 hours prior to the infectious blood meal. The blood meal consisted of 1∶1 100% swine-packed RBC (Innovative Research, USA) and a fresh ZIKV suspension, at a final concentration of 7.5 Log10 tissue culture infectious dose50/mL ((Log_10_TCID_50_/ml). Adenosine Triphosphate (Fermentas, USA), at a final concentration of 3 mM, was added to the blood meal as a phagostimulant. Mosquitoes were fed with an infectious blood meal that was warmed to 37°C using a Hemotek membrane feeding system (Discovery Workshops, Lancashire, United Kingdom) housed. After thirty minutes, mosquitoes were cold anesthetized and fully engorged females were transferred to 300 ml ca. paper cups and were maintained in an environmental chamber (Sanyo, Japan) at 29°C and 70–75% RH with a 12 h/12 h L∶D cycle and provided with 10% sugar/vitamin B complex *ad libitum*. All experiments were carried out in an arthropod containment level 2 (ACL-2) facility.

### Mosquito processing

To determine the ZIKV infection and dissemination rates in *Ae. albopictus*, 12 mosquitoes were sampled daily from day one to seven, and subsequently on day 10 and 14 post infection (pi). Saliva was collected using the forced salivation technique as previously described [27] with modification. The proboscis of each mosquito, with its legs and wings, was inserted into a micropipette tip containing 10 µl of M199 and allowed to salivate. After 45 minutes, the Medium 199 containing the saliva from each mosquito was transferred into microcentrifuge tubes containing 100 µl of M199. The midgut and salivary glands of each mosquito were processed as described by Li and colleagues [26]. Briefly, the midgut and the salivary glands were homogenized using stainless steel grinding balls (Retsch, Germany) in a MM301 mixer mill (Retsch, Germany) set at frequency of 12/sec for 1 min. The supernatant of the homogenate was applied in the viral titre assay. All dissecting needles were dipped in 80% ethanol and cleaned before being re-used. All experiments were conducted inside an ACL-2 facility.

### Quantitative reverse transcriptase-polymerase chain reaction (qRT-PCR) assay

Total RNA was isolated from saliva using the QIAamp Viral Mini Kit (Qiagen, Germany) following manufacturer's recommendations. ZIKV in saliva was detected using a one-step real-time reverse transcriptase-polymerase chain reaction (qRT-PCR) as previously described [10].

### Tissue Culture Infectious Dose_50_ (TCID_50_) assay

Viral titres from the midgut and salivary glands were determined with tissue culture infectious dose_50_ assay, an endpoint dilution technique, using Vero cells as described by Higgs et al. [28].

### Data analysis

The infection rate at each sampling day was determined by the percentage of infected midguts, while dissemination rate was calculated by dividing the number of infected salivary glands by the total number of mosquitoes with infected midguts. On the other hand, transmission rate was calculated by dividing the number of positive saliva by the number of infected salivary glands. Kolmogorov-Smirnov tests indicated that the data did not conform to conditions of normality, hence non-parametric analyses were performed. Kruskal-Wallis tests were used to determine differences in viral titres in midguts and salivary glands between days post-infection. When a significant difference was detected, Mann-Whitney *U*-tests were performed to determine which day differed. All analyses were performed in Minitab.

## Results

### Oral susceptibility of *Ae. albopictus* to ZIKV

From day 3-pi onwards, when blood had been completely digested, all midguts were positive for ZIKV, except for day 7- and 10-pi (Table 1). The presence of viable ZIKV in the salivary glands was first observed on day 3-pi in three mosquitoes. By day 5-pi, half of the mosquitoes sampled showed disseminated infection. From day 7-pi onwards, all mosquitoes were found to have ZIKV in their salivary glands (Table 1).

**Table 1:** Infection, dissemination and transmission rates for *Ae. albopictus* orally fed with ZIKV and held at 29°C at various days post-infection.

Days post- infection	Infection rate		Dissemination rate		Transmission rate	
	No. positive MG (number sampled)	Percent	No. positive SG (number sampled)	Percent	No. positive saliva (number sampled)	Percent
3	12 (12)	100	3 (12)	25	0 (3)	0
4	12 (12)	100	7 (12)	58.3	1 (7)	14.3
5	12 (12)	100	6 (12)	50	2 (6)	33.3
6	12 (12)	100	9 (12)	75	4(9)	44.4
7	11 (12)	91.7	11(11)	100	8 (11)	72.7
10	10 (12)	83.3	10(10)	100	10(10)	100
14	12 (12)	100	12(12)	100	12(12)	100

MG = midgut; SG = salivary gland.

Transmission was first observed on day 4 after the infectious blood meal and transmission rates were observed to increase at each sampling days. By day 10-pi onwards, ZIKV RNA was found in all saliva tested (Table 1).

### ZIKV midgut and salivary gland titres

**Figure 1:**
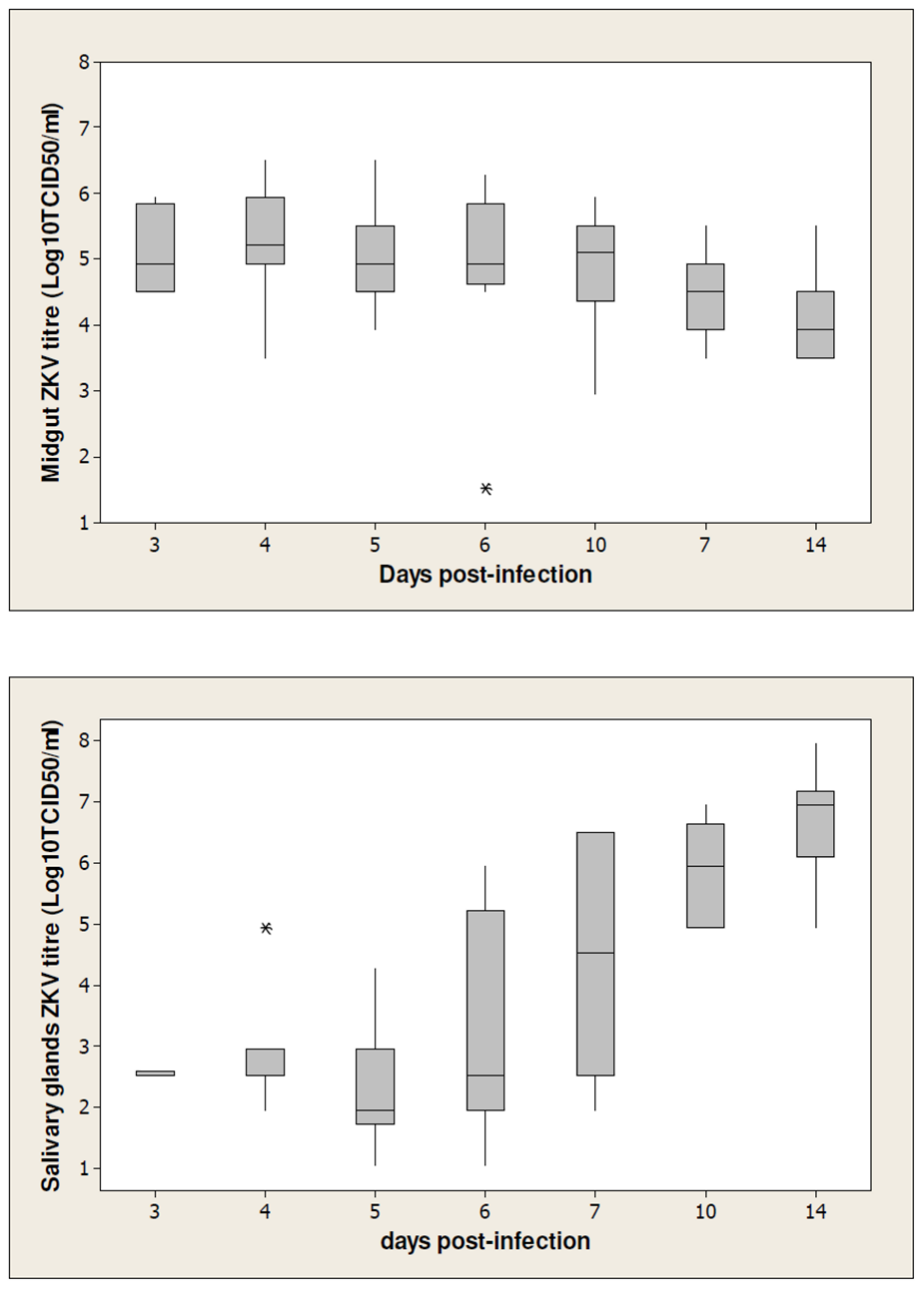
Box plots of ZIKV midgut and salivary glands titres at different days post-infection. Each box represents the median (horizontal bar), the interquartile range (box) and the full range (“whiskers”). Outliers are denoted by asterisk (*).

Figure 1 presents ZIKV midgut and salivary gland titres at different days pi. A significant difference in midgut ZIKV titres was observed between different days pi (Kruskal-Wallis test, *P* = 0.017). Midgut viral titres at days 3-, 4-, and 5-pi (>5.15 Log_10_TCID_50_/ml) were found to be significantly higher when compared to viral titre at day 14-pi (Mann-Whitney test, *P*<0.05) (Figure 1a). From days 6-pi onwards, viral titres fluctuated between 4.94 Log_10_TCID_50_/ml to 4.21 Log_10_TCID_50_/ml with no significant difference observed (Kruskal-Wallis test: *P* = 0.81).

In contrast, increasing ZIKV titre was observed in the salivary glands of *Ae. albopictus* (Figure 1b) (Kruskal-Wallis test, *P*<0.0001). Although the differences in ZIKV salivary gland titres from day 3-pi (2.54 Log_10_TCID_50_/ml) to day 6-pi (3.26 Log_10_TCID_50_/ml) were comparable (Mann-Whitney test, *P*≥0.09), the viral load increased significantly (Mann-Whitney test, *P*<0.05) by day 10-pi onwards (>5.96 Log_10_TCID_50_/ml).

## Discussion

Emerging and re-emerging mosquito-borne diseases are considered to be major threats to global health in both developing and developed countries. Their tendency of spreading outside their known geographic range and causing large scale epidemics has been clearly demonstrated during the recent global epidemic of CHIKV [29].

Zika is a neglected tropical disease, and like CHIKV, interest in ZIKV epidemiology was limited until recently, when its high epidemic potential was demonstrated during a large-scale outbreak in the Pacific Island of Yap in 2007 [10], [11], [13], [30]. During the outbreak that lasted four months, more than 70% of the island's population was affected [11]. Like many vector-borne diseases, the absence of vaccines and specific treatment against ZIKV means prevention and control relies heavily on vector control. Therefore, key information such as the identity of the vector, its bionomics, distribution, and density are needed in order to develop and implement sound mosquito control program. To date, little is known about the vectors of ZIKV outside Africa, except for *Ae. aegypti*.

The overlapping geographic distribution of *Ae. albopictus* with that of ZIKV has stimulated our interest to determine the potential of this mosquito to transmit ZIKV. Furthermore, the widespread distribution of *Ae. albopictus* in Singapore and large-scale local outbreaks of chikungunya in 2008–09 attest to the country's vulnerability to emerging mosquito-borne diseases [31]. The potential of these diseases to be established locally is accentuated by the country's reputation as a popular commercial and tourist hub, high dependency on migrant workers, tropical climate, dense human population, and the presence of potential mosquito vectors. Recently, we have shown the potential for ZIKV outbreak in Singapore by *Ae. aegypti* [26]. The study showed local *Ae. aegypti* are highly susceptible to ZIKV, with a short extrinsic incubation period (EIP) of five days.

Our current study showed that Singapore's *Ae albopictus* mosquitoes are susceptible to ZIKV, with high dissemination and transmission rates observed. By day 4-pi, 58% (*n* = 7/12) of the infected mosquitoes have disseminated infection and of these, three (43%) had ZIKV in their saliva. By day 7-pi, all infected mosquitoes are capable of transmitting the virus. A short EIP and high ZIKV salivary gland titres were also observed when we infect our local *Ae. aegypti* with ZIKV [26]. In that study, it took five and ten days post infectious blood meal to achieve a 62% and 100% dissemination rate, respectively. However, it does not mean that *Ae. albopictus* is more susceptible to ZIKV than *Ae. aegypti*, rather it could be due the amount of virus used to infect *Ae. aegypti* was lower (6.95 Log_10_ TCID50/mL) compared to the current study (7.52 Log_10_ TCID50/ml).

Although *Ae. albopictus* has long been a suspected vector of ZIKV [32], to our knowledge, this is the first report on the potential of this mosquito species to transmit ZIKV. However, further studies (such as entomological surveillance in endemic areas) are needed to validate our findings to ascertain the vectorial status of *Ae. albopictus* for ZIKV in nature especially when both *Ae. Aegypti* and *Ae. albopictus* are geographically sympatric. Nonetheless, data from our previous [26] and current studies suggest that both *Ae. aegypti* and *Ae. albopictus* will have significant roles in the transmission of ZIKV, should the virus be introduced in Singapore or in places where these mosquitoes abound. Recent phylogenetic analysis has identified two major lineages of ZIKV, an African and Asian lineage [17]. The strain used in our current study is the prototype MR766 Ugandan strain belonging to the former lineage. It will also be very interesting to study the strains belonging to the Asian lineage; unfortunately, at the time of the study, we only had access to the prototype strain.

Given the vulnerability of Singapore to ZIKV and presence of the virus in neighbouring countries, the Environmental Health Institute screened febrile cases not attributable to DENV and CHIKV for ZIKV and other arboviruses. To date, none was found positive for arboviruses other than DENV and CHIKV. Based on the information gathered from this study, the threat of ZIKV can be addressed by existing dengue and chikungunya control program being implemented in Singapore.

## References

[1] Paupy C, Delatte H, Bagny L, Corbel V, Fontenille D (2009) *Aedes albopictus*, an arbovirus vector: from the darkness to the light. Microbes Infect 11: 1177–1185. 19450706 10.1016/j.micinf.2009.05.005

[2] Gasperi G, Bellini R, Malacrida AR, Crisanti A, Dottori M, et al A new threat looming over the Mediterranean basin: emergence of viral diseases transmitted by *Aedes albopictus* mosquitoes. PLoS Negl Trop Dis 6: e1836. 10.1371/journal.pntd.0001836 PMC3459824 23029593

[3] Caminade C, Medlock JM, Ducheyne E, McIntyre KM, Leach S, et al Suitability of European climate for the Asian tiger mosquito *Aedes albopictus*: recent trends and future scenarios. J R Soc Interface 9: 2708–2717. 10.1098/rsif.2012.0138 PMC3427500 22535696

[4] Guillaumot L, Ofanoa R, Swillen L, Singh N, Bossin HC, et al Distribution of *Aedes albopictus* (Diptera, Culicidae) in southwestern Pacific countries, with a first report from the Kingdom of Tonga. Parasit Vectors 5: 247. 23130961 10.1186/1756-3305-5-247 PMC3497854

[5] Benedict MQ, Levine RS, Hawley WA, Lounibos LP (2007) Spread of the tiger: global risk of invasion by the mosquito *Aedes albopictus* . Vector Borne Zoonotic Dis 7: 76–85. 17417960 10.1089/vbz.2006.0562 PMC2212601

[6] Knudsen AB (1995) Global distribution and continuing spread of *Aedes albopictus* . Parassitologia 37: 91–97. 8778670

[7] Hawley WA (1988) The biology of *Aedes albopictus* . J Am Mosq Control Assoc Suppl 1: 1–39. 3068349

[8] Tsetsarkin KA, Vanlandingham DL, McGee CE, Higgs S (2007) A single mutation in chikungunya virus affects vector specificity and epidemic potential. PLoS Pathog 3: e201. 18069894 10.1371/journal.ppat.0030201 PMC2134949

[9] Lambrechts L, Scott TW, Gubler DJ (2010) Consequences of the expanding global distribution of *Aedes albopictus* for dengue virus transmission. PLoS Negl Trop Dis 4: e646. 20520794 10.1371/journal.pntd.0000646 PMC2876112

[10] Lanciotti RS, Kosoy OL, Laven JJ, Velez JO, Lambert AJ, et al (2008) Genetic and serologic properties of Zika virus associated with an epidemic, Yap State, Micronesia, 2007. Emerg Infect Dis 14: 1232–1239. 18680646 10.3201/eid1408.080287 PMC2600394

[11] Duffy MR, Chen TH, Hancock WT, Powers AM, Kool JL, et al (2009) Zika virus outbreak on Yap Island, Federated States of Micronesia. N Engl J Med 360: 2536–2543. 19516034 10.1056/NEJMoa0805715

[12] Kuno G, Chang GJ, Tsuchiya KR, Karabatsos N, Cropp CB (1998) Phylogeny of the genus Flavivirus. J Virol 72: 73–83. 9420202 10.1128/jvi.72.1.73-83.1998 PMC109351

[13] Hayes EB (2009) Zika virus outside Africa. Emerg Infect Dis 15: 1347–1350. 19788800 10.3201/eid1509.090442 PMC2819875

[14] Kuno G, Chang GJ (2007) Full-length sequencing and genomic characterization of Bagaza, Kedougou, and Zika viruses. Arch Virol 152: 687–696. 17195954 10.1007/s00705-006-0903-z

[15] Dick GW, Kitchen SF, Haddow AJ (1952) Zika virus. I. Isolations and serological specificity. Trans R Soc Trop Med Hyg 46: 509–520. 12995440 10.1016/0035-9203(52)90042-4

[16] Simpson DI (1964) Zika Virus Infection in Man. Trans R Soc Trop Med Hyg 58: 335–338. 14175744

[17] Haddow AJ, Williams MC, Woodall JP, Simpson DI, Goma LK (1964) Twelve Isolations of Zika Virus from *Aedes* (Stegomyia) *africanus* (Theobald) Taken in and above a Uganda Forest. Bull World Health Organ 31: 57–69. 14230895 PMC2555143

[18] Akoua-Koffi C, Diarrassouba S, Benie VB, Ngbichi JM, Bozoua T, et al (2001) [Investigation surrounding a fatal case of yellow fever in Cote d'Ivoire in 1999]. Bull Soc Pathol Exot 94: 227–230. 11681215

[19] McCrae AW, Kirya BG (1982) Yellow fever and Zika virus epizootics and enzootics in Uganda. Trans R Soc Trop Med Hyg 76: 552–562. 6304948 10.1016/0035-9203(82)90161-4

[20] Fagbami AH (1979) Zika virus infections in Nigeria: virological and seroepidemiological investigations in Oyo State. J Hyg (Lond) 83: 213–219. 489960 10.1017/s0022172400025997 PMC2129900

[21] Weinbren MP, Williams MC (1958) Zika virus: further isolations in the Zika area, and some studies on the strains isolated. Trans R Soc Trop Med Hyg 52: 263–268. 13556872 10.1016/0035-9203(58)90085-3

[22] Marchette NJ, Garcia R, Rudnick A (1969) Isolation of Zika virus from *Aedes aegypti* mosquitoes in Malaysia. Am J Trop Med Hyg 18: 411–415. 4976739 10.4269/ajtmh.1969.18.411

[23] Heang V, Yasuda CY, Sovann L, Haddow AD, Travassos da Rosa AP, et al Zika virus infection, Cambodia, 2010. Emerg Infect Dis 18: 349–351. 10.3201/eid1802.111224 PMC3310457 22305269

[24] Cornet M, Robin Y, Adam C, Valade M, Calvo MA (1979) [Comparison between experimental transmission of yellow fever and zika viruses in *Aedes aegypti*]. Cah ORSTOM ser Ent med et Parasitol 17: 47–53.

[25] Boorman JP, Porterfield JS (1956) A simple technique for infection of mosquitoes with viruses; transmission of Zika virus. Trans R Soc Trop Med Hyg 50: 238–242. 13337908 10.1016/0035-9203(56)90029-3

[26] Li MI, Wong PS, Ng LC, Tan CH (2012) Oral susceptibility of Singapore *Aedes* (Stegomyia) *aegypti* (Linnaeus) to Zika virus. PLoS Negl Trop Dis 6: e1792. 22953014 10.1371/journal.pntd.0001792 PMC3429392

[27] Anderson SL, Richards SL, Smartt CT (2010) A simple method for determining arbovirus transmission in mosquitoes. J Am Mosq Control Assoc 26: 108–111. 20402359 10.2987/09-5935.1 PMC2858320

[28] Higgs S, Olson KE, Kamrud KI, Powers AM, Beaty B (1997) Viral expression systems and viral infections in insects. ; Beard CB, Louis C, editors. London: Chapman and Hall. 459–483 p.

[29] Ng LC, Hapuarachchi HC (2010) Tracing the path of Chikungunya virus–evolution and adaptation. Infect Genet Evol 10: 876–885. 20654736 10.1016/j.meegid.2010.07.012

[30] Mackenzie JS, Williams DT (2009) The zoonotic flaviviruses of southern, south-eastern and eastern Asia, and Australasia: the potential for emergent viruses. Zoonoses Public Health 56: 338–356. 19486319 10.1111/j.1863-2378.2008.01208.x

[31] Ng LC, Tan LK, Tan CH, Tan SS, Hapuarachchi HC, et al (2009) Entomologic and virologic investigation of Chikungunya, Singapore. Emerg Infect Dis 15: 1243–1249. 19751586 10.3201/eid1508.081486 PMC2815960

[32] Olson JG, Ksiazek TG, Suhandiman, Triwibowo (1981) Zika virus, a cause of fever in Central Java, Indonesia. Trans R Soc Trop Med Hyg 75: 389–393. 6275577 10.1016/0035-9203(81)90100-0

